# Late saphenous vein graft rupture presenting as a compressive mediastinal pseudoaneurysm

**DOI:** 10.21542/gcsp.2026.17

**Published:** 2026-04-30

**Authors:** Dimitrios Afendoulis, Sotirios Tsalamandris, Flora Tsakirian, Nikolaos Tsiamis, Georgios Benetos, Kostas Tsioufis, Konstantinos Toutouzas

**Affiliations:** 1 Unit of Structural and Valvular Heart Diseases, 1st Department of Cardiology, NKUA, ‘Hippokration’ General Hospital of Athens, Greece

## Abstract

Late saphenous vein graft (SVG) pseudoaneurysm is a rare but life-threatening complication of coronary artery bypass graft surgery. An 80-year-old man presented with dyspnea and chest pain two decades after CABG (LIMA-LAD, SVG-RCA). Coronary angiography revealed SVG occlusion with contrast extravasation and mass formation adjacent to the right heart chambers. CT angiography confirmed a 39 mm mediastinal pseudoaneurysm from proximal SVG rupture, compressing the right heart chambers and lung, with consequent left ventricular dysfunction (EF 45%), elevated pulmonary pressures, and orthostatic hypotension. Following Heart Team discussion, the patient underwent successful pseudoaneurysm resection with right coronary artery revascularization using a left radial artery graft. Symptoms and cardiac function improved at one-month follow-up. This case highlights the importance of clinical suspicion for SVG pseudoaneurysm in patients with prior CABG, and the central role of CT angiography and multidisciplinary decision-making in management.

## Case presentation

An 80-year-old man with history of hypertension, diabetes, coronary artery disease and coronary artery bypass graft 20 years ago (LIMA-LAD, SVG-RCA) was referred for coronary angiography after experiencing dyspnea and chest pain consistent with angina for the past month (NYHA Class-II). Medication consisted of statin, metformin, GLP-1 receptor agonist, aspirin, tamsulosin and olmesartan.

**Figure 1.**
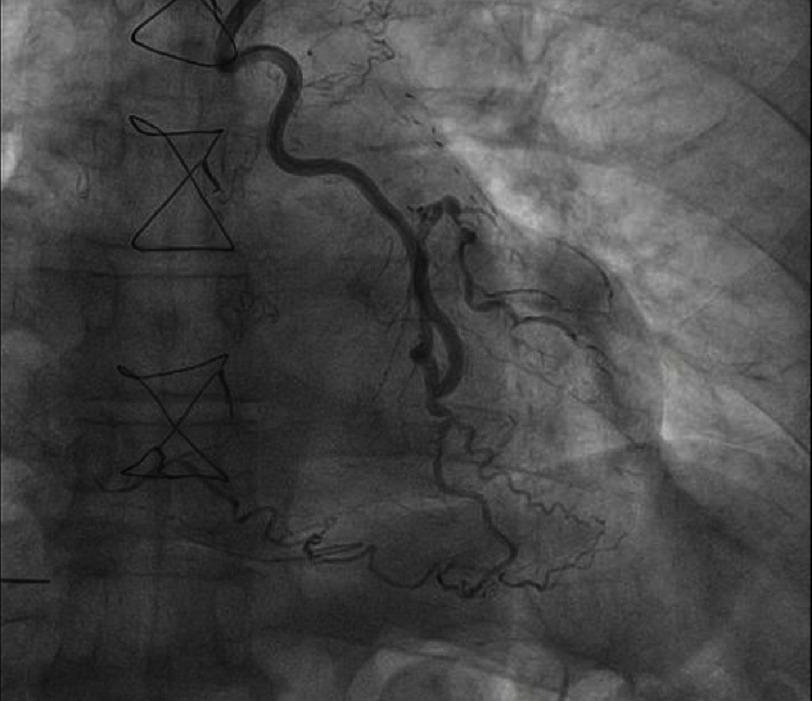
Coronary angiography depicting the patent LIMA to the LAD.

**Figure 2.**
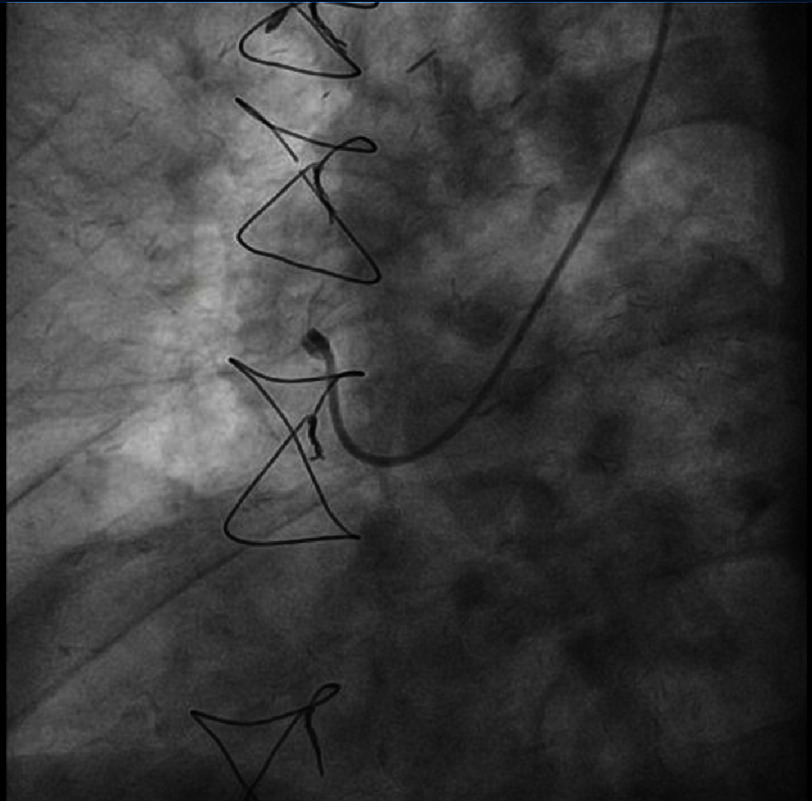
Coronary angiography demonstrating the occluded saphenous vein graft.

**Figure 3.**
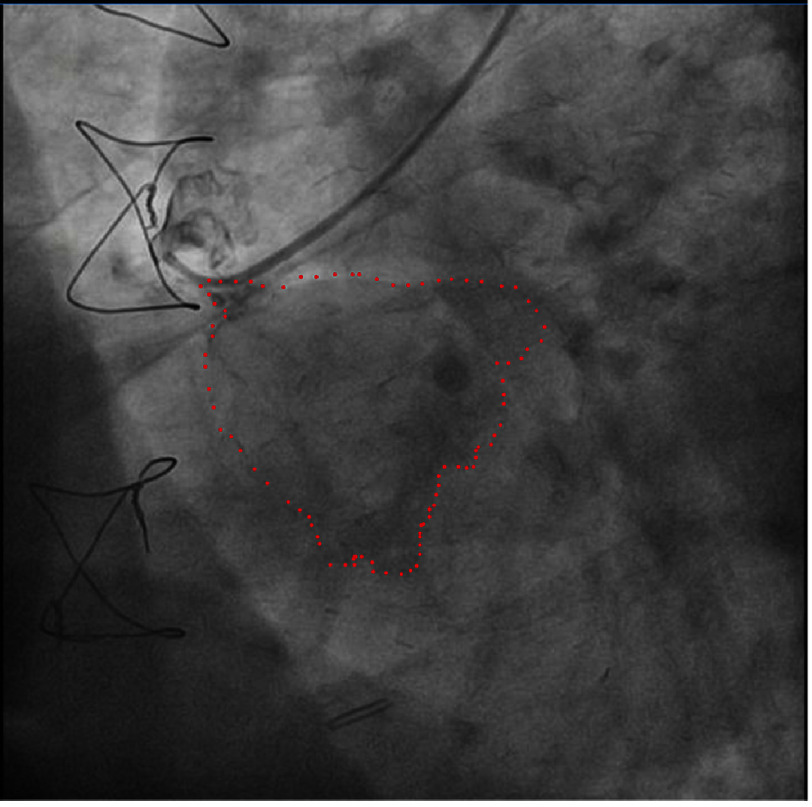
Coronary angiography depicting the presence of contrast flow out of the vessel with the formation of a mass in contact with the right heart chambers (red dots).

The angiogram showed a patent left internal mammary artery graft to the left anterior descending artery, proximal occlusion of the right coronary artery (Figures 1 and 2). Notable during the contrast infusion to the saphenous vein graft was the presence of contrast flow out of the vessel with subsequent formation of a mass in contact with the right heart chambers (Figure 3, red dots).

## Further diagnostic evaluation

**Figure 4.**
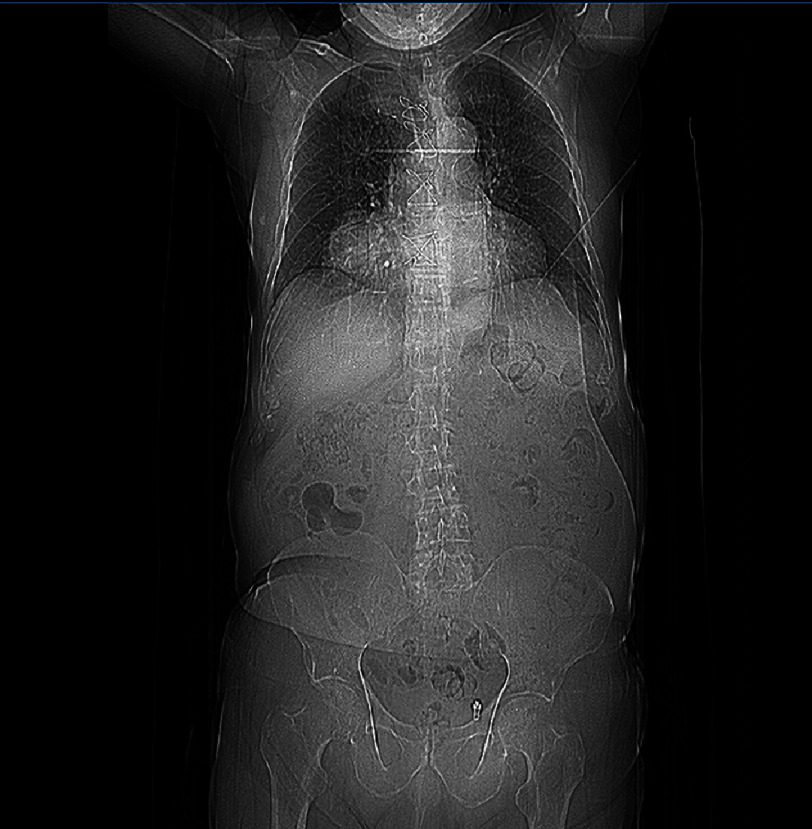
Chest X-ray showing an enlargement of the right cardiac silhouette.

Given these findings, our patient underwent further work-up. On examination, orthostatic hypotension was observed. Laboratory testing revealed a creatinine value of 1.5 mg/dL, a high sensitivity troponin-I value of 35 ng/L and an elevated NT-proBNP value of 1,300 pg/mL and an HbA1C value of 7.1%, with no other significant findings. Chest radiography demonstrated an enlargement of the right cardiac silhouette (Figure 4). Transthoracic echocardiography revealed impaired left ventricular function with mildly reduced left ventricular ejection fraction (LVEF 45%) due to hypokinesis of the inferior wall of the left ventricle (due, in turn, to the proximal occlusion of the right coronary artery and saphenous vein occlusion) and pulmonary hypertension, but no pericardial effusion.

**Figure 5.**
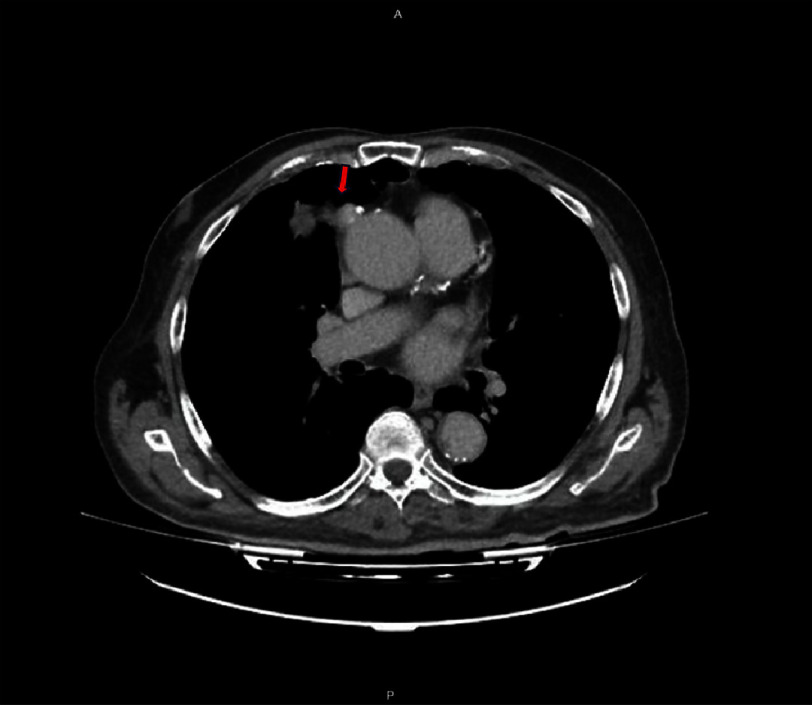
Computed tomography angiography of the thorax with intravenous contrast, demonstrating rupture of the saphenous vein graft proximally and formation of a pseudoaneurysm-red arrow.

**Figure 6.**
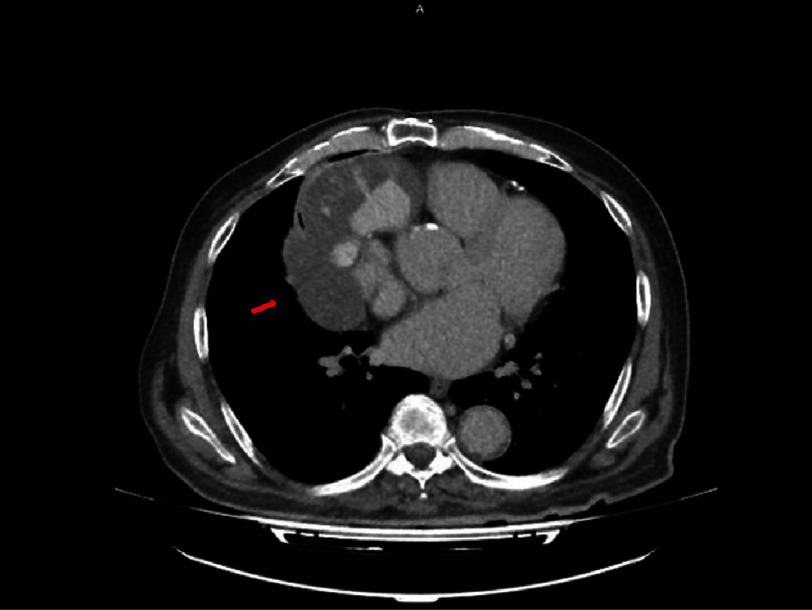
Computed tomography angiography of the thorax with intravenous contrast, demonstrating a mediastinum mass consistent with a pseudoaneurysm of maximal diameter of 39 mm (red arrow) due to rupture of the saphenous vein graft proximally. The mass was in contact with the right heart chambers, pressing also the right lung.

In light of these results and the patient’s symptoms, CT angiography of the chest with intravenous contrast was carried out and demonstrated a mediastinal mass consistent with a proximal rupture of the saphenous vein graft and formation of a pseudoaneurysm measuring 39 mm in diameter (Figures 5 and 6). The mass was compressing the right heart chambers and right lung, accounting for the patient’s symptoms.

## Decision-making

The large size of the pseudoaneurysm which was pressing the right lung and right heart chambers was a possible explanation of patient’s symptoms. Dyspnea and chest pain were attributed to this and elevated pulmonary pressures as well. Moreover, the orthostatic hypotension could partially be explained by the reduced preload of the left ventricle due to right heart pressure and was improved after cessation of olmesartan.

Taking into consideration the pseudoaneurysm’s large size, compressive effects on the right heart chambers and right lung, the presence of symptoms, and high risk of rupture, the Heart Team decided on open surgical repair. Given the pseudoaneurysm’s large size and the total occlusion of the saphenous vein graft and the right coronary artery proximally, the percutaneous approach was not judged suitable. The patient underwent a successful operation with pseudoaneurysm resection and arterial graft using the left radial artery. Postoperative recovery was normal.

## Follow-up

The patient presented with improvement of symptoms and cardiac function on the echocardiography during his one-month follow-up. Our patient was scheduled for a coronary computed tomography angiography after six months to confirm the patency of the grafts.

## Discussion

Late saphenous vein graft failure due to rupture and pseudoaneurysm formation is a rare complication of coronary artery bypass grafting. Several mechanisms may lead to this complication including structural vulnerability of vein grafts, which have thinner walls than arterial grafts, characterised by remodelling and dilation of the medial smooth muscle and elastin layers upon exposure to systemic pressures and elevated wall shear stress.

Surgical factors like anastomotic trauma or technical imperfections (such as inadequate ligation of tributaries) can predispose to later wall dehiscence. Vein grafts can undergo degenerative changes and accelerated atherosclerosis, with mechanisms including intimal proliferation and hyperplasia, foam cell infiltration, extracellular matrix degradation, and chronic inflammation, which progressively weaken the graft wall.

Cytokines (IL-1, IL-6, TNF-α) and matrix metalloproteinases from macrophages degrade collagen and elastin, facilitating pseudoaneurysm formation. Postoperative infection or chronic localized inflammation can compromise structural integrity and promote wall rupture^1^.

Clinical presentation is variable and non-specific, with symptoms including dyspnea and chest pain determined largely by the pseudoaneurysm’s size and its relationship to adjacent cardiac, pulmonary, and vascular structures. A high index of clinical suspicion, informed by the patient’s history, is essential for diagnosis and should prompt cross-sectional imaging, particularly CT angiography^2–4^.

Fistulous communication with other heart chambers or the pericardium is possible, leading to hemodynamic compromise or tamponade. Such pseudoaneurysms carry a risk of rupture, with catastrophic complications, and thus intervention is crucial upon diagnosis. Surgical repair remains the treatment of choice. The procedure typically consists of resection of pseudoaneurysm with concomitant coronary revascularization, often using arterial graft to restore myocardial perfusion.

In selected high-risk surgical candidates, percutaneous closure has emerged as a viable alternative. Various closure devices have been successfully used, especially when the pseudoaneurysm has a narrow neck and the graft segment distal to the pseudoaneurysm is occluded or functionally irrelevant^1–3^. Covered stents deployed to seal the pseudoaneurysm while preserving distal coronary flow and may be used in cases of leaking pseudoaneurysm, high surgical risk, or emergent cases.

Multiple reports show their effective exclusion of SVG pseudoaneurysms with covered stents, including recurrent pseudoaneurysms treated with sequential stent deployment under IVUS or OCT guidance. Long-term follow-up indicates durable results with thrombus resolution and graft patency maintenance.

Another alternative remains catheter-based coil embolization into the pseudoaneurysm to promote thrombosis, typically reserved for pseudoaneurysms with small distal flow territories. Small, localized pseudoaneurysms may be treated with percutaneous thrombin injection. Finally, occlusion with Amplatzer Vascular Plugs percuaneously, has proven useful for larger, non-critical or thrombosed grafts.

Conservative medical management, with optimisation of antiplatelet and cardiovascular treatment (statins, angiotensin receptor inhibitors and beta-blockers) may be selected in cases of small, asymptomatic pseudoaneurysms in patients with high surgical risk and patent distal vessels^5,6^. Key considerations for mode of therapy selection remain the size and growth of the pseudoaneurysm (intervention generally recommended when greater than one cm or symptomatic lesions), the risk of rupture or embolization, distal vessel territory and necessity of preserving coronary perfusion, patient’s frailty, comorbidities and surgical risk and expertise in percutaneous options. The decision should be made with a multidisciplinary approach in the context of the heart team. According to these data, surgical repair remains the gold standard for large pseudoaneurysms causing symptoms due to structure compression or requiring revascularization, as in our case. Percutaneous covered stent intervention, or closure with vascular plugs or coils is a viable alternative in high-risk or emergency cases and may be repeated in cases of reoccurrence. Finally, conservative management with follow-up imaging is feasible for small, stable pseudoaneurysms in patients unsuitable for surgery or percutaneous procedures^7–9^.

## Conclusions

Late saphenous vein graft rupture with pseudoaneurysm formation is a rare but potentially life-threatening complication of coronary artery bypass grafting. Clinical presentation is often non-specific, and a high index of suspicion is essential for timely diagnosis. Surgical repair remains the gold standard for large, symptomatic pseudoaneurysms, particularly when concomitant revascularization is required. Percutaneous closure techniques represent a viable alternative in high-risk or emergency cases and may be repeated in the event of recurrence. Management decisions should always be made within a multidisciplinary heart team framework, taking into account patient-, procedural-, and pseudoaneurysm-related factors.

## Funding

No funding was received for this study.

## Conflicts of interest

The authors declare that they have no conflicts of interest.

## Ethics

Patient’s informed consent for publication of the manuscript was obtained.
